# Impact of Leucocyte Depletion and Prion Reduction Filters on TSE Blood Borne Transmission

**DOI:** 10.1371/journal.pone.0042019

**Published:** 2012-07-31

**Authors:** Caroline Lacroux, Daisy Bougard, Claire Litaise, Hugh Simmons, Fabien Corbiere, Dominique Dernis, René Tardivel, Nathalie Morel, Stephanie Simon, Séverine Lugan, Pierrette Costes, Jean Louis Weisbecker, François Schelcher, Jacques Grassi, Joliette Coste, Olivier Andréoletti

**Affiliations:** 1 UMR INRA ENVT 1225, Interactions Hôte Agent Pathogène, Ecole Nationale Vétérinaire de Toulouse, Toulouse, France; 2 UPR CNRS 1142, R&D TransDiag, EFS Pyrénées –Méditerranée, Montpellier, France; 3 AHVLA Weybridge, ASU, New Haw, Addlestone, Surrey, United Kingdom; 4 EFS Nord de France, Production Department, Lille, France; 5 EFS Bretagne, Production Department, Rennes, France; 6 CEA, Service de Pharmacologie et d'Immunoanalyse, IBiTec-S, DSV, CEA/Saclay, Gif sur Yvette, France; 7 INRA Domaine de Langlade, Pompertuzat, France; Creighton University, United States of America

## Abstract

The identification in the UK of 4 v-CJD infected patients thought to be due to the use of transfused Red Blood Cell units prepared from blood of donors incubating v-CJD raised major concerns in transfusion medicine. The demonstration of leucocyte associated infectivity using various animal models of TSE infection led to the implementation of systematic leuco-depletion (LD) of Red Blood cells concentrates (RBCs) in a number of countries. In the same models, plasma also demonstrated a significant level of infectivity which raised questions on the impact of LD on the v-CJD transmission risk. The recent development of filters combining LD and the capture of non-leucocyte associated prion infectivity meant a comparison of the benefits of LD alone versus LD/prion-reduction filters (LD/PR) on blood-borne TSE transmission could be made. Due to the similarity of blood/plasma volumes to human transfusion medicine an experimental TSE sheep model was used to characterize the abilities of whole blood, RBCs, plasma and buffy-coat to transmit the disease through the transfusion route. The impact of a standard RBCs LD filter and of two different RBCs LD/PR prototype filters on the disease transmission was then measured. Homologous recipients transfused with whole-blood, buffy-coat and RBCs developed the disease with 100% efficiency. Conversely, plasma, when intravenously administered resulted in an inconstant infection of the recipients and no disease transmission was observed in sheep that received cryo-precipitated fraction or supernatant obtained from infectious plasma. Despite their high efficacy, LD and LD/PR filtration of the Red Blood Cells concentrate did not provide absolute protection from infection. These results support the view that leuco-depletion strongly mitigates the v-CJD blood borne transmission risk and provide information about the relative benefits of prion reduction filters.

## Introduction

Transmissible spongiform encephalopathies (TSE), or prion diseases, are fatal neurodegenerative disorders naturally occurring in sheep (scrapie), cattle (bovine spongiform encephalopathy - BSE), and humans (Creutzfeldt-Jakob disease - CJD).

In humans, CJD is a rare disease (about 1 case per million and per year) that usually occurs as either a sporadic (s-CJD), familial or genetic form. Despite their relative rareness, there have been hundreds of iatrogenic CJD cases, following corneal and dura-mater grafts, the use of extractive pituitary hormones or the contamination of deep intracranial electrodes that have been reported over the last 50 years [1]. In that context, the hypothesis of an inter-human blood borne transmission of CJD has been carefully considered and actively surveyed by health authorities. Although low levels of infectivity could be detected in different rodent experimental models, large scale retrospective epidemiological studies failed to demonstrate any association between the occurrence of CJD and transfusion of blood/plasma or the administration of plasma derived products [2]–[5]. These elements led to the contention that blood borne CJD inter-human transmission risk was negligible.

In 1996, a new form of CJD, named variant CJD (v-CJD) was identified in humans. Variant CJD was demonstrated to be due to the same agent that causes BSE in cattle and its emergence in humans was established to be the consequence of a dietary exposure to BSE contaminated products [6], [7]. Variant CJD differs from the other described human CJD diseases in many aspects. In particular, an early and persistent accumulation of TSE agent is described in lymphoid tissues of v-CJD infected patients whereas in the patients affected with other CJD forms, the infectious agent is mostly restricted to the central and peripheral nervous system [8].

The presence of the v-CJD agent in lymphoid tissues combined with the detection of leucocyte associated infectivity in TSE rodent models [9], [10], raised major concerns with regards to a potential blood borne v-CJD transmission risk.

Since then, four probable v-CJD transmissions through transfusion were reported in the UK, all the patients having received Red Blood Cell units prepared from donors who developed symptoms of v-CJD 17 months to 3,5 years after donation [11], [12]. Despite the declining trend of v-CJD incidence, which passed a peak in the year 2000 in UK, and the control of the dietary source of exposure to the BSE agent, the prevalence of the v-CJD in the exposed population remains unknown. In the UK, a retrospective study of stored tonsils and appendix tissues found three positive appendix samples in 12 674 i.e. about 1/4000 though with wide confidence intervals [13]. More recently a modelling study estimated that the number of the blood borne v-CJD cases in the UK (277 cases predicted) might exceed the number of cases caused by dietary exposure to BSE observed so far (176 cases) [14]. Although the accuracy of these estimates continues to be refined, it indicates that the v-CJD blood-borne transmission risk cannot be considered negligible.

Before any v-CJD blood borne transmission had been recognized, some countries implemented measures intended to prevent its potential transmission by blood products. Amongst them, France (1998), Ireland, UK, Portugal (1999) and Canada (2000) introduced systematic leuco-depletion (LD) for labile blood products and in 2001 also for plasma in France.

However, the possible benefits of leuco-depleted red blood cell concentrates (LD-RBCs) use with regards to the v-CJD transmission risk remains unclear. Data obtained in rodent models indicated that only 40% of the infectivity seemed to be associated with the white blood cells (WBC), while plasma would contain 60% [9]. Based on these results, and the fact that around 10 mL of plasma remains in each RBC unit, it could be expected that leuco-depletion alone might be unable to provide a significant prion infectivity reduction. In that context, one company (MacoPharma, Tourcoing, France) developed a prion removal device (P-Capt) to reduce the plasma associated infectivity from RBCs by incorporating a prion-specific resin into a biocompatible filter. This resin was reported to provide a greater than 3 log_10_ reduction of infectivity in RBCs spiked with infectious brain homogenate and a reduction to the limit of detection (>1 log_10_) of the blood endogenous infectivity in rodent TSE models [15], [16]. However, the relevance of the ‘spiking’ model used to evaluate the performances of Prion reduction filters is debatable as the TSE infectivity in blood of a v- CJD patient could have different biological properties to that of spiked brain homogenate. Additionally, the assessments of the potential performance of LD and prion reduction filters rely on ‘infectious load’ as measured by intracerebral inoculation in rodents, that does not reflect the specifics of the transfusion exposure route; *i.e.* the administration of large numbers of living cells and volume intravenously to the recipient [17]. Therefore, the impact of combined LD and prion reduction filters on blood borne v-CJD transmission risk still remains to be accurately assessed.

Variant CJD’s pathogenesis displays features very similar to natural classical scapie in sheep. In both cases, infection occurs following oral exposure and an early preclinical accumulation of the infectious agent in lymphoid tissues is described [18]–[20]. The relative similarity in size between human and sheep allows the transfusion of blood volumes that are relevant to human medicine. Additionally, whereas blood groups exist in sheep, the absence of pre-existing antibodies raised against blood groups antigens allows transfusion with low risk of adverse effects [21]. In early 2000, transmission of both experimental BSE and natural scrapie were reported to occur following transfusion of whole blood collected in asymptomatic incubating sheep [22], [23]. In these models, whole blood and various blood components (RBC, plasma, Buffy coat) were shown to transmit TSE when transfused to healthy recipients [24], [25]. Although it cannot be assumed in any animal model TSE agents in blood behave identically to that in v-CJD in humans, sheep TSE models are today considered as relevant sources of information for assessing v-CJD blood borne transmission risk.

In this study, using an experimental scrapie in sheep infection model [25], [26], we investigated the relative abilities of whole blood, RBCs, plasma and Buffy-coat prepared from preclinical infected and/or clinically affected animals to transmit disease through the transfusion route. Blood components were prepared following methods used in human transfusion medicine and the impact of a standard LD filter and of two different formats of LD/PR filters on the disease transmission rate caused by RBCs transfusion was measured. Our results demonstrate the high efficacy of LD filters and provide information for assessment of the relative benefits of prion reduction filters.

## Methods

### Ethics Statement

All animal experiments have been performed in compliance with our institutional and French national guidelines, in accordance with the European Community Council Directive 86/609/EEC. Experimental protocol was approved by the INRA Toulouse/ENVT ethics committee.

### Sheep

The VRQ/VRQ sheep were produced in a biosecure unit from a flock that was originally sourced from New Zealand by AHVLA UK. This DEFRA funded TSE free flock’ is a unique source of VRQ/VRQ animals that can be considered safe for classical scrapie [27].

The animals included in our experiments were imported in France and housed in a dedicated scrapie free farm situated at 30 km from the experimental facilities before their use in experiments. Dedicated staff, having no contact with the infectious animals, bred them.

### PG127 Classical Scrapie Oral Inoculation Sheep Model

The classical scrapie isolate was derived from experimentally VRQ/VRQ affected sheep (PG127 isolate). 6–10 months old TSE-free sheep (AHVLA- Weybridge- UK) were orally challenged with 2 g equivalent of brain material (1% brain homogenate in glucose). This isolate has been previously end-point titrated by intracerebral route in tg338 mice (10^6.8^ ID_50_/g) [28]. Animals were then observed until the occurrence of clinical signs (around 200 days post inoculation) and culled when exhibiting locomotor signs of the disease that impaired their feeding capacities. After culling each sheep was necropsied and a variety of lymphoid tissues (spleen, third eye-lid, mesenteric lymph node, prescapular lymph node, tonsil) were sampled in addition to the CNS for PrP^Sc^ detection.

### Sheep Genotyping

In all cases, PrP genotype was obtained by sequencing the Exon 3 of the *Prnp* gene as previously described [29].

### Combination Filters for Leucodepletion/Prion Reduction (LD/PR) of RBCs

Two devices developed by different manufacturers for filtering human RBCs were evaluated in this study: Asahi KASEI combination filter (Oita, Japan) and Pall Medical Leukotrap Affinity Plus (East Hills, NY, USA). Both devices complied with the standard requirements for RBCs processing in transfusion medicine. The exact composition/structure of these devices is patented and then protected by commercial confidence. Both devices were demonstrated by manufacturers to achieve a greater than 3 log_10_ reduction of infectivity using human RBCs spiked with 263 K hamster scrapie.

### Blood Collection and Preparation

The blood collection from the 15 infected sheep was performed in the UMR INRA/ENVT 1225 experimental facilities using Fenwal (Lake Zurich, USA) disposables R7542 top and bottom (TAB) collection bags. Whole blood units were shipped overnight to the EFS Nord de France for the preparation of the blood components according to the French Transfusion Service processes.

**Figure 1:**
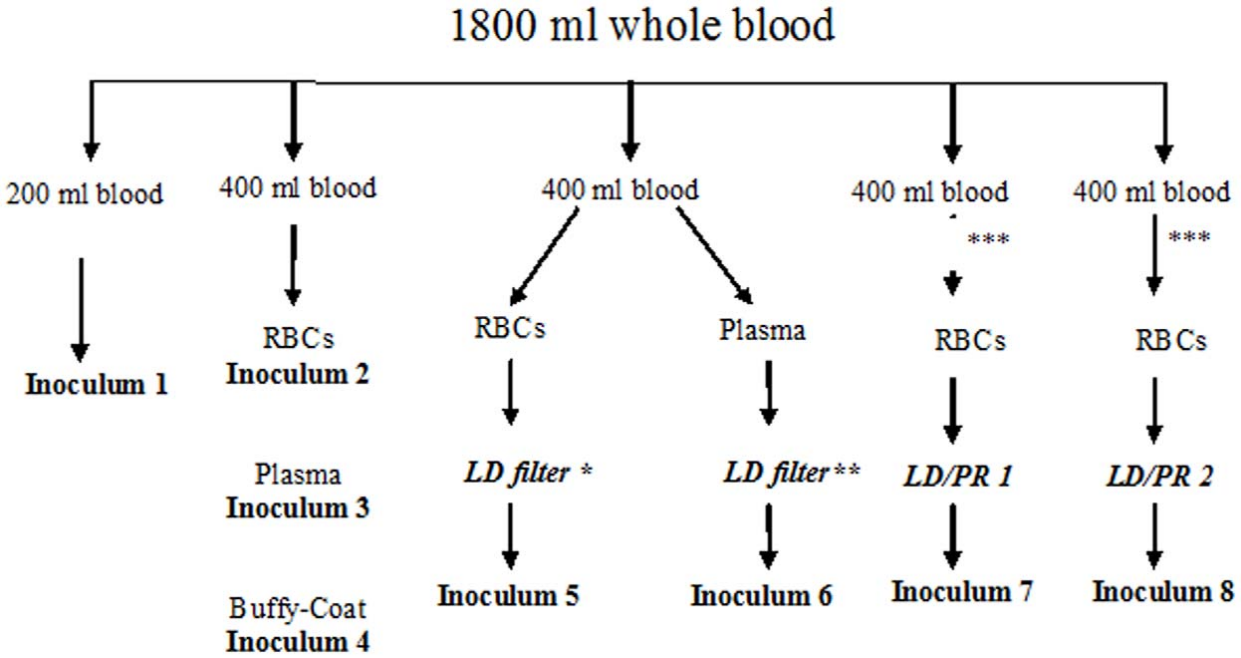
Design of blood products preparation and filtration process. 600 mL to 700 mL of whole blood were collected at the late stage of preclinical incubation from 15 VRQ/VRQ sheep orally inoculated with PG127 scrapie isolate. Blood from 3 sheep were pooled in a single pouch in order to obtain a volume higher than 1800 mL. Each pool was split into 5 parts that were individually processed according to the standard procedure applied in transfusion medicine. Obtained fractions were transfused or intravenously administered to TSE free VRQ/VRQ sheep, within five days following the blood collection. LD: leuco-depletion – LD/PR: leuco-depletion/Prion reduction. *:SEPACELL PURE RC (ASAHI). **: Plasma Filter Fenwal. ***: plasma for titration and cryoprecipitation study.

Blood from 3 sheep were pooled to obtain a volume higher than 1800 mL : the 3 collected units were mixed in a transfer bag (3000 mL, MacoPharma, Mouvaux, France) and then split into 5 Fenwall R7542 TAB collection bags : 1 unit of 200 mL and 4 units of 400 mL (Figure 1).

The 200 mL whole blood unit represents Inoculum 1.

The 400 ml units (x4) were centrifuged at 3640 rpm for 12 minutes (acceleration and still) at 20°C in a Jouan LR5-22 centrifuge in order to separate the plasma, the buffy-coat and the red cells concentrate (RBCs).

The first unit of 400 ml was centrifuged and then separated in plasma, buffy-coat and RBCs (Inoculums 2–3–4).

The second unit of 400 ml was centrifuged, to obtain plasma and RBC. RBCs and plasma were then leuco-depleted using the Fenwal R7542 disposal : Sepacell® Pure RC filter from ASAHI for RBCs and Fenwal plasma filter for plasma (Inoculums 5–6).

The third and fourth units of 400 ml were also centrifuged and separated in plasma, buffy-coat and RBCs but only the RBCs were leuco-depleted, using LD/PR filter from either manufacturer 1 or manufacturer 2 (Inoculums 7–8).

### Plasma Cryoprecipitation

Cryo-precipitation is the first step of the standard procedure applied to extract coagulation factors from plasma. Non leuco-reduced plasma corresponding to the third and fourth units (Inoculum 7 and 8 preparation lines in Figure 1) were kept frozen at −30°C for 3 months. They were then submitted to a standard cryoprecipitation procedure at UMR INRA/ENVT 1225 laboratory. Then 200 mL of frozen plasma were incubated at 1°C (permanent temperature monitoring) overnight in a cryostat and then transferred in 50 mL conic tubes on ice, centrifuged (4000 g) for 1 H at 1°C. Supernatant was then collected by pipetting before −80°C storage of both supernatant and pellet.

### Transfusion of Whole Blood and Blood Components

Recipient sheep were anesthetized by IV administration of Ketamine/Diazepam (respectively 2 mg and 0.1 mg per Kg) and a venal catheter (16 G) was installed (jugular vein). Whole blood (200 mL), plasma (150–200 mL) and RBCs (about 200 mL) were transfused over a 30–40 min period. Buffy-coat samples were gently re-suspended in a 200 mL sterile pouch of isotonic (5%) glucose before administration over a 20–30 min period. Transfusion of the whole blood was performed within the 3 hours following blood collection. In the experiment aiming at the assessment of LD and LD/PR filter efficacy, transfusion of the recipients with the different components was performed within the 5 days following the blood collection.

In the plasma titration experiment (by transfusion route), adequate whole plasma volumes were transferred into a new pouch or gently re-suspended in a sterile 5% glucose solution (QSP 200 mL), before IV administration in recipients. Similarly, cryoprecipitate and cryprecipitation supernatants were re-suspended in 5% glucose solution (200 mL) and immediately administered intravenously by gravity over a 30 min period.

### Sheep Monitoring and Necropsy

Sheep were housed in a category 2 animal facility. Donor sheep and recipients were kept separated. Contact controls (n = 5) were housed in the same pen as transfusion recipients sheep. These controls were housed throughout the experiment and none displayed any TSE symptoms. Animals were daily monitored. When displaying symptoms that could impair their capacity to feed, the animals were euthanized by an intravenous injection of T61 (Intervet) followed by exsanguination. Transfused animals that did not display clinical signs after 400–450 days were also euthanized. At the end of the complete experiment, contact controls were also euthanized.

Each animal was systematically necropsied and a panel of tissues was collected (brain, spleen, mesenteric lymph node, tonsil, third eyelid, prescapular lymph node, ileum). The samples were divided in two parts, the first one was formalin fixed for PrP^Sc^ Immunohistochemistry investigation and the other was snap frozen at −80°C for Western Blot abnormal PrP detection.

### Abnormal PrP Western-blot Detection (WB)

A Western blot kit (TeSeE Western Blot, Bio-Rad) was used following the manufacturer’s recommendations. For each sample 250 µL of 10% brain homogenate were submitted to PrP^Sc^ extraction. The obtained pellet was denaturated in Laemmli’s buffer (15 µL) before being loaded neat or diluted on a 12% acrylamide gel, before electrophoresis and blotting. Immunodetection was performed using Sha31 monoclonal antibody which recognizes the 145–152 sequence of PrP (YEDRYYRE), conjugated to horseradish peroxidase (at 0,06 µg per mL). Peroxidase activity was revealed using ECL substrate (Pierce) [30].

### PrP^Sc^ Immunohistochemistry (IHC)

This method was performed as previously described [31]. PrP^Sc^ IHC detection was performed using 8G8 antibody raised against human recombinant PrP protein and specifically recognising the 95–108 amino acid sequence (SQWNKP) of the PrP protein.

## Results

Fifteen TSE free sheep were orally inoculated with 2 g of the same PG127 classical scrapie isolate and 600 to 700 mL of blood was collected in citrate dextrose pouches from each sheep between 160 and 170 days post inoculation (dpi). At this time point, the animals showed no clinical signs but subsequently developed them and were killed after a mean incubation period of 231+/−15 dpi. *Post mortem* analysis confirmed the accumulation of abnormal PrP in both their lymphoid tissues and central nervous system (CNS) (Table 1). Previous experiments carried out using the same model indicated that blood collected from 130 dpi in PG127 orally challenged sheep transmitted the disease with 100% efficacy in transfusion recipients [25], and also demonstrated that the transfusion of 200 µL of blood from asymptomatic infected donor sheep transmitted prion disease with 100% efficiency (Andreoletti et al, in press).

**Table 1:** Composition of the 5 sheep whole blood pools used for preparation of labile blood products.

	Collection date	Donor		PrP^Sc^	
		ID	Incubationperiod	CNS	LRS
**Pool 1**	161 dpi	**D1**	228 dpi	+	+
		**D2**	234 dpi	+	+
		**D3**	249 dpi	+	+
**Pool 2**	163 dpi	**D4**	225 dpi	+	+
		**D5**	217 dpi	+	+
		**D6**	221 dpi	+	+
**Pool 3**	168 dpi	**D7**	209 dpi	+	+
		**D8**	228 dpi	+	+
		**D9**	248 dpi	+	+
**Pool 4**	169 dpi	**D10**	209 dpi	+	+
		**D11**	256 dpi	+	+
		**D12**	226 dpi	+	+
**Pool 5**	170 dpi	**D13**	222 dpi	+	+
		**D14**	250 dpi	+	+
		**D15**	244 dpi	+	+

Each pool was obtained by mixing 600–700 mL of whole blood collected in 3 VRQ/VRQ sheep orally inoculated with PG127 classical scrapie isolate. After blood collection (collection date) each donor was monitored till the occurrence of clinical signs. Donors were culled when displaying locomotor difficulties that could impair their abilities to feed (incubation period). TSE was confirmed by detection of abnormal PrP in lymphoid tissues (LRS) and central nervous system (CNS) using immunohistochemistry and Western Blot.

After collection, whole blood from three individuals was pooled in order to obtain pools of 1800 mL (Figure 1). These pools were then divided into different bags before being further processed. 200 mL of whole blood were kept untouched (inoculum 1). 400 mL were processed in plasma, RBCs and Buffy-Coat (inoculums 2, 3 and 4).

The three others whole blood units (each containing 400 mL) were prepared in plasma and RBCs. One plasma and RBCs units were leuco-depleted using LD filters routinely used to prepare blood components (inoculum 5 and 6). The two last RBCs were processed using LD/PR filters that were developed by the two different manufacturers (inoculum 7 and 8). All steps were carried out using standard equipment and procedures employed by transfusion service and the two LD/PR filters were used under the supervision of the manufacturers’ staff.

At different points in the processes, samples were analyzed in order to characterize the efficacy of LD (Table 2). The preparation of the RBC (elimination of the leuco-platelet fraction) showed a significant reduction of leukocytes and all three tested filters reduced the leucocytes numbers to less than 10^6^ leukocytes per RBCs unit, which complies with the current requirements in transfusion medicine.

**Table 2:** Efficacy of the Leuco-depletion and Leuco-depletion/Prion reduction filters on sheep Red Blood Cell concentrates.

	Whole blood	RBC – Leuco depletion filter		RBC leuco- depletion/prion reduction filter 1		RBC leuco- depletion/prion-reduction filter 2	
		Pre-filtration	Post-filtration	Pre-filtration	Post-filtration	Pre-filtration	Post-filtration
**Pool 1 Leucocytes (10^6^/mL)**	6.3	1.06	<0.005	2.35	<0.005	1.39	<0.005
**Pool 2 Leucocytes (10^6^/mL)**	1.73	0.55	<0.005	0.47	<0.005	0.39	<0.005
**Pool 3 Leucocytes (10^6^/mL))**	3.75	2.97	0.005	0.49	<0.005	0.55	<0.005
**Pool 4 Leucocytes (10^6^/mL)**	1.82	0.52	<0.005	0.38	<0.005	0.33	<0.005
**Pool 5 Leucocytes (10^6^/mL)**	4.75	0.44	<0.005	0.17	<0.005	0.4	<0.005

Each whole blood pool was constituted by mixing 600–700 mL from 3 VRQ/VRQ sheep orally inoculated with PG127 classical scrapie isolate. Aliquots of 400 mL of whole blood were processed to prepare Red Blood Cell concentrate (RBC). RBCs were then filtered using either a leuco-depletion filter (Sepacell pure RC –Asahi) or two Leucodepletion depletion/Prion reduction filters (LD/PR). Filtered RBC volumes varied between 155 and 190 mL.

Signs of clinical TSE infection were observed in all VRQ/VRQ sheep that received whole blood, Buffy-coat or RBCs (Table 3). No clinical disease was observed in recipients that were transfused with LD or LR/PR filtered RBCs. However, in two VRQ/VRQ sheep, one who received a LD RBCs (pool 4) and one who received the LD/PR RBCs (pool 3), analysis of the tissues after culling (400 dpi) revealed the presence of abnormal PrP in lymphoid tissues and/or in central nervous system. The absence of clinical disease occurrence after 400 days incubation suggests an exposure to very low titre of infection. None of the five sheep that received RBCs treated with the second LD/PR, displayed detectable abnormal PrP accumulation in their tissues.

**Table 3:** TSE occurrence in VRQ/VRQ TSE free sheep transfused with blood labile products prepared from Scrapie infected sheep.

	Pool 1			Pool 2			Pool 3			Pool 4			Pool 5		
		PrP^Sc^			PrP^Sc^			PrP^Sc^			PrP^Sc^			PrP^Sc^	
	Incubation	LRS	CNS	Incubation	LRS	CNS	Incubation	LRS	CNS	Incubation	LRS	CNS	Incubation	LRS	CNS
**Whole blood**	199	+	+	203	+	+	234	+	+	178	+	+	195	+	+
**RBCs**	197	+	+	209	+	+	196	+	+	226	+	+	191	+	+−
**Plasma**	>400	−	−	195	+	+	>400	−	−	222	+	+	227	+	+
**Buffy-Coat**	179	+	+	175	+	+	201	+	+	174	+	+	183	+	+
**Leuco- depleted Plasma**	>400	−	−	>400	−	−	>400	−	−	227	+	+	>400	−	−
**Leuco-depleted RBCs**	>400	−	−	>400	−	−	>400	−	−	>400	+	−	>400	−	−
**Leuco depletion/** **prion reduction** **filter 1 RBCs**	>400	−	−	>400	−	−	>400	−	−	>400	−	−	>400	−	−
**Leuco depletion/** **prion reduction filter 2 RBCs**	>400	−	−	>400	−	−	>400	+	+	>400	−	−	>400	−	−

Each whole blood pool was constituted by mixing 700 mL from 3 VRQ/VRQ sheep orally inoculated with PG127 classical scrapie isolate. Aliquots of whole blood were processed according to the design described in Figure 1. Obtained products were then transfused in VRQ/VRQ TSE free recipient sheep. Recipients were monitored for clinical TSE occurrence. Clinically affected animals were culled when displaying locomotor difficulties that could impair their abilities to feed. The remaining apparently healthy recipients were killed 400 days after transfusion. Systematic detection of abnormal PrP (using both immunohistochemistry and western-blot) in lymphoid tissues (LRS) and central nervous system (CNS) was carried out in recipients as to establish their status.

Only 3 out the 5 fresh plasma (about 250 mL – pools 2, 4 and 5) recipient sheep developed disease (Table 3), and one of the LD plasma recipients was found positive.

In parallel, an end-point titration experiment was carried out in TSE free sheep using four out of these five non leuco-depleted plasma: 200 mL, 20 mL, 2 mL of plasma (pools 1 to 4 - Figure 1) were intravenously administrated to recipients. Transmission was observed in sheep that received 200 mL of plasma from pool 2 and 4 (Table 4) but none of the animals that received lower volume developed the disease or displayed detectable PrP deposition in their tissues after culling (450 dpi).

**Table 4:** TSE occurrence in VRQ/VRQ TSE free sheep transfused with frozen plasma and plasma fractions prepared from Scrapie infected sheep.

	Pool 1			Pool 2			Pool 3			Pool 4		
		PrP^Sc^			PrP^Sc^			PrP^Sc^			PrP^Sc^	
	Incubation	LRS	CNS	Incubation	LRS	CNS	Incubation	LRS	CNS	Incubation	LRS	CNS
**Plasma 200 mL**	>450	−	−	208	+	+	>450	−	−	201	+	+
**Plasma 20 mL**	>450	−	−	>450	−	−	>450	−	−	>450	−	−
**Plasma 2** **mL**	>450	−	−	>450	−	−	>450	−	−	>450	−	−
**Cryoprecipitate** **(200 mL plasma)**	>450	−	−	>450	−	−	>450	−	−	>450	−	−
**Supernatant** **(200** **mL plasma)**	>450	−	−	>450	−	−	>450	−	−	>450	−	−

Whole blood pool was constituted by mixing 600–700 mL from 3 VRQ/VRQ sheep orally inoculated with PG127 classical scrapie isolate. Aliquots of blood were processed as described in Figure 1. For each pool, plasma obtained from Inoculum 7 and 8 preparation lines were stored at −30°C. Decreasing amount of plasma (200 mL, 20 mL and 2 mL) were then transfused to VRQ/VRQ recipients. In parallel, 200 mL of plasma were submitted to standard cryoprecipitation procedure. Both supernatant and cryoprecipitate were intravenously administered to VRQ/VRQ recipient sheep. Recipients were monitored for clinical TSE occurrence. Clinically affected animals were culled when displaying locomotor difficulties that could impair their abilities to feed. The remaining apparently healthy recipients were killed 450 days after transfusion. Systematic detection of abnormal PrP (using both immunohistochemistry and western-blot) in lymphoid tissues (LRS) and central nervous system (CNS) was carried out in recipients as to establish their status.

The lack of disease transmission observed in sheep that received 20 mL of plasma, combined with the observation that all 5 RBCs transmitted the disease support the contention that the 10 mL of residual plasma that remains associated to RBCs play a minor role in the disease transmission risk.

Finally, 200 mL of the plasma pools (1 to 4) used in the titration experiment were submitted to cryo-precipitation. The supernatant and precipitate obtained from each of the plasma were re-suspended in 5% glucose solution (200 mL) and intravenously administrated to VRQ/VRQ recipient sheep (Table 4). Although disease transmission had been observed in sheep that received 200 mL of pool 2 and 4 plasmas but, no clinical signs or abnormal PrP accumulation (culling at 450 dpi) were observed in sheep that received those cryoprecipitates or supernatants.

On *post mortem* none of the 5 VRQ/VRQ contact controls, who were housed with transfusion recipients, displayed abnormal PrP deposition in their lymphoid tissues or CNS, demonstrating that lateral or environmental cross contamination is unlikely as an explanation for disease in any of the recipients.

## Discussion

On the basis of the relative infectious titre in plasma, as assessed in rodent models, LD has been considered as a potentially beneficial at reducing the risk of v-CJD transmission associated with RBCs transfusion but this risk redution was insufficient measure [15].

The results obtained in this experiment using the PG127 sheep infection model indicated that the transfusion of standard RBCs, prepared using 400 mL of blood, transmitted the disease with 100% efficiency compared to only one out of the five recipients of the LD-RBCs. This efficacy of disease transmission by the transfusion of LD-RBCs (that corresponded to 400 mL of whole blood) was lower than the one reported in the same model using 200 µL of whole blood [17]. Together these results clearly demonstrate that within the limits of our model, LD of RBCs provided a major reduction of the disease transmission.

To date, 4 v-CJD cases have been identified in patients who received non leuco-depleted RBCs [32]. In the UK, three of the recipients who contracted the disease developed clinical signs after incubation periods varying from 6 to 8.5 years [11], [33], [34]. Leuco-reduction was implemented more than 11 years ago in a large number of European countries and since then, no transfusion-transmitted v-CJD case has been reported in patients who received LD RBCs. Although it cannot be assumed that such patients will not develop v-CJD in the future due to the potential length of incubation, but this and the data that we reported here reinforce the view that LD has reduced the potential risk of v-CJD blood borne transmission.

In the UK (n = 24/176) and France (n = 3/25) patients that had developed v-CJD had been blood donors. Plasma from these patients entered in the composition of several hundreds of batches of plasma products and in France the number of patients that were intravenously treated with those products exceeded 50 000 [32]. Amongst people that received plasma derived products, those who are treated for chronic disorders like haemophilia or immunodeficiency are potentially more exposed than other patients. Recently one preclinical v-CJD case was reported in the UK in a haemophilic patient who died from a condition unrelated to v-CJD [35]. The patient had been treated with several batches of UK sourced clotting factors before 1999. The patients’ treatment had included one batch of FVIII that was manufactured using plasma from a donor who went on to develop symptoms of v-CJD six months after donating his plasma in 1996. However, it still remains unclear at this stage whether this v-CJD case should be attributed to plasma derived products or to another source.

Our results indicate that, in the PG127 scrapie model, plasma displayed limited capacity to transmit the disease by transfusion. This observation concurs with the results recently reported in both BSE infected sheep and Chronic Wasting Diseases infected cervids [24], [36]. The results also support the view that leuco-depletion filters further reduce the risk of transmitting TSE by plasma.

The lack of disease transmission observed in sheep that received the plasma cryoprecipitate or the cryo-precipitation supernatant is surprising as the results obtained in rodents TSE models demonstrated that during cryo-precipitation process plasma infectivity split between cryoprecipitate and supernatant without significant reduction of the global infectivity amount [9]. Considering the low ability of the plasma to transmit the disease by transfusion that was observed in this work, it could be hypothesized that such infectivity splitting might explain the incapacity of both cryoprecipitate and supernatant to infect recipient. Further experiments will be needed to test this hypothesis. It also seems important to confirm that the limited infectivity of the plasma that was observed in PG127 infected sheep is also valid in sheep infected with other TSE agents (BSE for instance). Beyond these limitations, the results obtained suggest that plasma derived products, in particular those prepared from leuco-reduced plasma might still display a limited risk to transmit the disease.

Based on the assumption that leuco-depletion would be insufficient to prevent v-CJD transmission by the transfusion of RBCs, several manufacturers developed blood prion-reduction filters. These filters were designed to capture TSE infectivity that would not be associated with leucocytes. The low infectious titre in whole blood (as measured by the intracereabral route in rodents) was a major limitation to assessing the efficacy of these filters [15], as the evaluations were mainly carried out using blood spiked with exogenous infectivity (generally brain homogenate). In this paradigm, the filters can reduce the infectivity load by 3 log_10_ [16], [37]–[39]. However, spiking with brain material is thought to be of limited relevance towards blood endogenous infectivity.

In this experiment, no clinical cases were observed in recipients transfused with LD/PR filtered RBCs but one apparently healthy recipient was found infected after culling, suggesting its exposure to low titre infectious material. Therefore, the comparison of the transmission rates obtained with the LD filter alone and the LR/LP filters, does not appear to support the view that prion-reduction filters had a significant additional beneficial effect on the disease transmission. However considering the limits of our experiment (only 5 recipients were transfused for each filtration device) and the fact that no infection was observed in animals that were transfused with RBC filtered with one of the two tested of LD/PR filter, additional experiments would be necessary to statistically assess the potential benefits of this device. If such experiment should be carried out, it would be critically important to determine whether the additional beneficial effect of the LD/PR filter could be due to its ability to achieve higher level of leuco-reduction (final number of leucocytes in RBC unit) than the one obtained by standard LD filter.

Currently, the distribution and level of infectivity in blood components of v-CJD affected patients remains undocumented. In that context, even if appropriate caution should be taken when interpreting the data, TSE animal models represent the main source of information for assessing v-CJD blood borne transmission risk. Under the hypothesis that the blood transfusion model in sheep represents a pertinent model for v-CJD blood borne TSE transmission, the results we obtained indicate that plasma is significantly less efficient than RBCs and buffy-coat to transmit the disease by the transfusion route. Our results also strongly support the view that RBCs and plasma leuco-reduction has a major impact on the mitigation of the v-CJD transmission risk in transfusion medicine and provide information to help with the assessment of the relative benefits of prion reduction filters.
